# Differences in maternal gene expression in Cesarean section delivery compared with vaginal delivery

**DOI:** 10.1038/s41598-020-74989-8

**Published:** 2020-10-20

**Authors:** Prachi Kothiyal, Keriann Schulkers, Xinyue Liu, Sahel Hazrati, Thierry Vilboux, Luis M. Gomez, Kathi Huddleston, Wendy S. W. Wong, John E. Niederhuber, Thomas P. Conrads, G. Larry Maxwell, Suchitra K. Hourigan

**Affiliations:** 1 Inova Health System, Falls Church, VA USA; 2 Inova Children’s Hospital, Falls Church, VA USA; 3 Persona Biomed, Inc., Alexandria, VA USA; 4 Women’s Service Line and the Women’s Health Integrated Research Center, Inova Health System, Falls Church, VA USA; 5 College of Health and Human Services, George Mason University, Fairfax, VA USA; 6 Departments of Surgery and Oncology, Johns Hopkins University School of Medicine, Baltimore, MD USA

**Keywords:** Gene expression profiling, Transcriptomics, Functional clustering

## Abstract

Cesarean section (CS) is recognized as being a shared environmental risk factor associated with chronic immune disease. A study of maternal gene expression changes between different delivery modes can add to our understanding of how CS contributes to disease patterns later in life. We evaluated the association of delivery mode with postpartum gene expression using a cross-sectional study of 324 mothers who delivered full-term (≥ 37 weeks) singletons. Of these, 181 mothers had a vaginal delivery and 143 had a CS delivery (60 with and 83 without labor). Antimicrobial peptides (AMP) were upregulated in vaginal delivery compared to CS with or without labor. Peptidase inhibitor 3 (PI3), a gene in the antimicrobial peptide pathway and known to be involved in antimicrobial and anti-inflammatory activities, showed a twofold increase in vaginal delivery compared to CS with or without labor (adjusted p-value 1.57 × 10^–11^ and 3.70 × 10^–13^, respectively). This study evaluates differences in gene expression by delivery mode and provides evidence of antimicrobial peptide upregulation in vaginal delivery compared to CS with or without labor. Further exploration is needed to determine if AMP upregulation provides protection against CS-associated diseases later in life.

## Introduction

While it is widely known that birthing a child is a physiologically stressful event, the research surrounding how it affects the expression of genes in both mothers and infants is still sparse. Cesarean section (CS) is now recognized as being a shared environmental risk factor associated with chronic immune diseases in the offspring^1–3^. At the cellular level, hematological and immunologic markers differ in the umbilical cord blood from those born by vaginal delivery (VD) compared with CS, with higher numbers of leukocytes, including neutrophils, monocytes and natural killer cells detected in VD^4,5^. Children born by CS have an increased risk for developing certain diseases later in life, such as asthma and allergies^2,3,6–9^, obesity^10–12^, connective tissue disorders^1^, inflammatory bowel disease^1,2^, type I diabetes mellitus ^13^ and childhood leukemia^14^. Mothers who have given birth by CS have also been found to have a higher risk for developing an autoimmune disease as compared to those who gave birth by VD^15,16^.

Surgery may induce gene expression changes related to adverse outcomes. A recent study that examined changes in gene expression after major thoracoabdominal surgery demonstrated that post-operatively, genes related to innate immunity and inflammation were upregulated while those related to adaptive immunity were downregulated^17^. One study thus far identified epigenetic changes in the genome that occurred surrounding birth; more specifically infants born by CS had an increase in DNA-methylation in leukocytes compared to those born by VD. It is not yet known if these epigenetic changes have a lasting effect on both mother and child^18^.

Gene expression analysis of peripheral whole blood can provide an in-vivo perspective into one’s response to physiological stressors and pathologic insults^19^. Furthermore, gene expression of patients with a variety of diseases correlates well with the status of the disease^20,21^. As such, in an attempt to explore if gene expression changes vary by method of delivery, RNA sequencing data was examined in mothers at time of delivery. We hypothesized that CS delivery (with or without labor) compared with VD is associated with gene expression changes in the mother, which could potentially contribute to disease patterns later in life in the mother and offspring.

## Results

### Demographic and clinical data

A total of 324 mothers with RNA sequencing available were included in the analysis. Of these, 181 had a VD and 143 had a CS delivery (60 with and 83 without labor) (Table 1). Mothers undergoing CS without labor were older than those undergoing VD or CS with labor (mean age CS without labor 34.3 years vs. CS with labor 31.4 years vs. VD 31.5 years; ANOVA p-value = 0.0002). Mothers undergoing CS without labor had a slightly lower gestational age at delivery than those having VD or CS with labor, although all deliveries were full term (mean gestational age CS without labor 38.8 weeks vs. CS with labor 39.1 weeks vs. VD 39.3 weeks; ANOVA p-value = 0.001). For mothers who had a CS, the indications for CS were also recorded (Table 1). For CS with labor, the top reason for CS was failure to progress or prolonged labor (N = 34) and for mothers undergoing CS without labor, the top reason was repeat CS (N = 67).

**Table 1:** Summary demographic data across delivery modes.

	Vaginal delivery (N = 181)	CS with labor (N = 60)	CS without labor (N = 83)	p-value
**Ethnicity**				**0.1**^**+**^
Not Hispanic or Latino	100	39	58	
Hispanic or Latino	60	12	17	
Declined	3	3	1	
Unknown	18	6	7	
**Race**				
White or Caucasian	102	38	54	
Asian	12	7	9	
Black or African American	6	0	2	
Declined	3	3	1	
Other/More than one Race	19	4	6	
Unknown	39	8	11	
Age (years)	**31.5** (30.8–32.2)	**31.4** (30.1–32.7)	**34.3** (33.0–35.5)	**0.0002**^**++**^
Body mass index (kg/m^2^)	**24.7** (24.1–25.4)	**25.9** (24.5–27.2)	**25.8** (24.6–27.0)	**0.1**^**++**^
Gestational age at delivery (weeks)	**39.3** (39.1–39.4)	**39.1** (38.7–39.5)	**38.8** (38.5–38.9)	**0.001**^**++**^
**Indications for CS**				
Repeat CS		12	67	
Failure to progress/prolonged labor		34	0	
Fetal distress		16	3	
Abnormal positioning		9	5	
Maternal health problem		5	8	
Cephalopelvic disproportion/fetal macrosomia		6	6	
Premature rupture of membranes		4	2	
Fetal defects		1	3	
Placental issue		0	2	
Oligohydramnios		1	1	
Polyhydramnios		1	1	
Cord prolapse		1	0	
Parental choice/elective		0	1	
Uterine issue		1	0	

Subject counts across self-reported race and ethnicities are included. Group average values for age, body mass index and gestational age are provided, and 95% confidence interval is included within parenthesis. For the two CS groups, aggregated counts are included for different indications for CS. It is to be noted that the sum of these counts exceeds total number of subjects in the group as multiple indications were recorded for each subject when applicable.

^+^p-value corresponds to Fisher’s Exact Test for counts.

^++^p-values correspond to one-way ANOVA test across the three delivery modes.

### CS (with or without labor) vs. vaginal delivery

**Figure 1:**
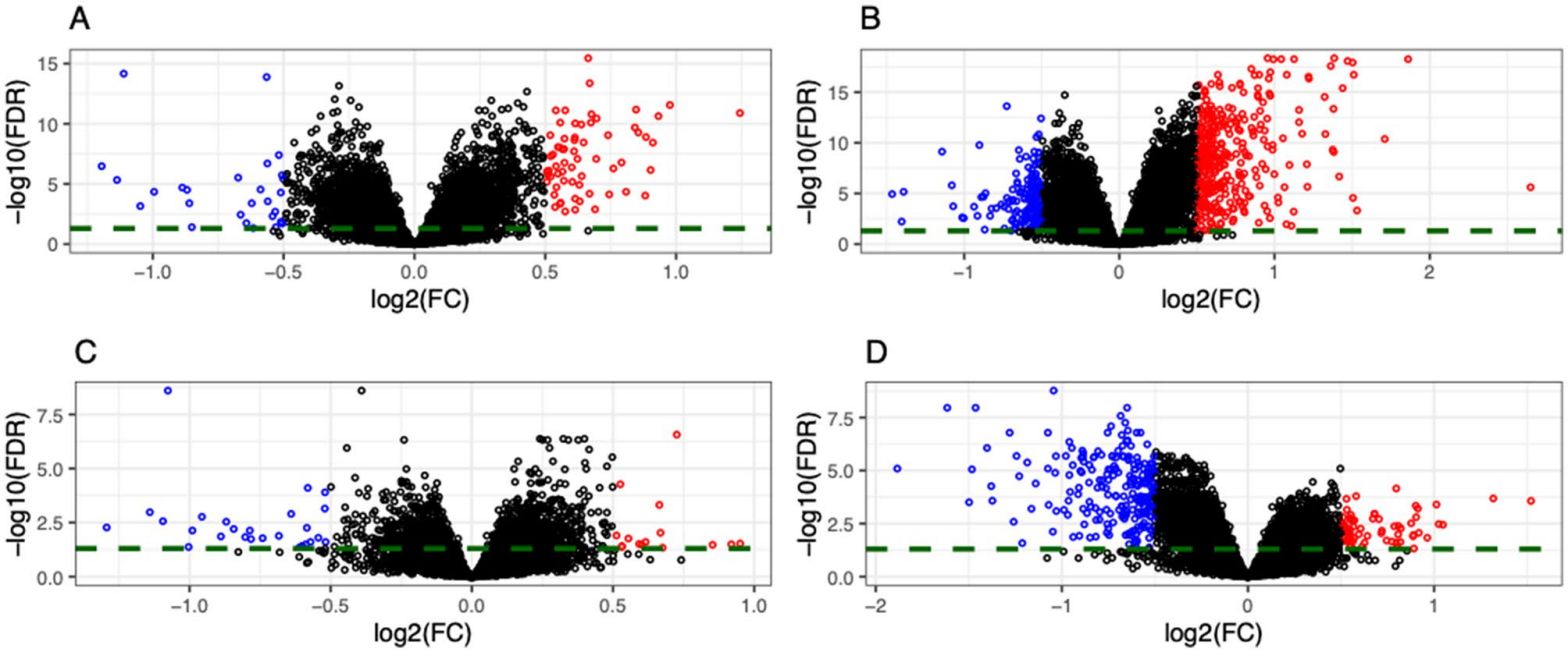
Volcano plots of differentially expressed genes in pairwise comparisons across delivery modes. Differentially expressed genes (DEG) are selected based on FDR ≤ 0.05 and absolute value of log_2_(fold-change) ≥ 0.5; genes with ≥ twofold-change are labeled in each plot. (**A**) DEGs (N = 91) in vaginal delivery vs. CS (with or without labor). Genes are colored based on upregulation (red; N = 64) or downregulation (blue; N = 27) in CS (with or without labor). (**B**) DEGs (N = 506) in vaginal delivery vs. CS with labor. Genes are colored based on upregulation (red; N = 343) or downregulation (blue; N = 163) in CS. (**C**) DEGs (N = 41) in vaginal delivery vs. CS without labor. Genes are colored based on upregulation (red; N = 15) or downregulation (blue; N = 26) in CS. (**D**) DEGs (N = 263) in CS with labor vs. without labor. Genes are colored based on upregulation (red; N = 60) or downregulation (blue; N = 203) in CS without labor. VD N = 181, CS with labor N = 60, CS without labor N = 83. *FDR* false discovery rate, *FC* fold-change, *CS* cesarean section, *VD* vaginal delivery.

Samples from CS (with or without labor, N = 143) vs. VD (N = 181) were compared. Ninety-one genes were found to be differentially expressed at FDR ≤ 0.05 and magnitude of log_2_(fold-change) ≥ 0.5 as highlighted in the volcano plot in Fig. 1A and listed in Supplementary Table S1. Of the differentially expressed genes, 27 (30%) were upregulated in VD. Antimicrobial peptides were the top pathway and included PI3, which was the top gene upregulated in VD (Supplementary Table S2). No genes were found to be differentially expressed between delivery modes in an independent analysis of prenatal samples from these mothers, thus confirming the changes did not exist before delivery (lowest FDR > 0.99).

### CS with labor vs. vaginal delivery

Samples from CS with labor (N = 60) vs. VD (N = 181) were compared. Using a cutoff for false discovery rate (FDR) ≤ 0.05 and magnitude of log_2_(fold-change) ≥ 0.5, 506 genes were found to be differentially expressed (Fig. 1B, Supplementary Table S3). Of the 506 differentially expressed genes, 163 (32%) were upregulated in VD and were enriched in the pathways listed in Table 2. Antimicrobial peptides and interferon signaling were among the top pathways and included the PI3 gene. Genes in neutrophil degranulation and interleukin signaling pathways were upregulated in CS with labor. No genes were found to be differentially expressed in a separate analysis of only prenatal samples (lowest FDR > 0.99).

**Table 2:** Top pathways for differentially expressed genes in CS (with labor) vs. vaginal delivery.

Pathway	Adjusted p-value	Group with upregulation	Genes
Antimicrobial peptides	0.008	VD	GNLY;DEFA4;SEMG1;DEFA3;PRTN3;CTSG; DEFA1;DEFA1B;PI3;ELANE;GZMH
Interferon alpha/beta signaling	0.008	VD	RSAD2;OAS3;MX1;IFIT1;USP18;OASL
Immunoregulatory interactions between a Lymphoid and a non-Lymphoid cell	0.009	VD	TRAV19;CD40LG;IGLV7-46;IGKV1-12; MADCAM1;KIR3DL2;IGLV7-43;SIGLEC1; SIGLEC8;KLRG1
Chemokine receptors bind chemokines	0.009	VD	CCL5;CXCR3;XCL2;CCR9;CXCR6
Interferon Signaling	0.01	VD	HERC5;RSAD2;OAS3;MX1;IFIT1; USP18; HLA-DQB2;OASL
Antiviral mechanism by IFN-stimulated genes	0.01	VD	HERC5;OAS3;MX1;IFIT1;USP18;OASL
Neutrophil degranulation	1.66 × 10^–9^	CS with labor	SERPINB10;MGST1;HP;SLC2A3;GPR84;RETN; LILRA3;FCAR;PLAC8;TRPM2;HK3;GM2A; ALOX5;CLEC5A;STOM;S100A12;CD59;CD36; GYG1;CD55;CD177;MGAM;SERPINB1;FCER1G; CR1;SERPINB2;GCA;ARG1;GPR97;C19orf59; ATP11B;MMP8;OLFM4;MAPK14;MMP9;CKAP4;CEACAM1;CLEC4D;VNN1;DLC1;SLCO4C1; S100P;CYSTM1;S100A9;CD44;S100A8
Interleukin-4 and Interleukin-13 signaling	0.004	CS with labor	SOCS3;ANXA1;BCL6;HGF;ALOX5;OSM;RHOU; CD36;JAK2;MMP9

All pathways containing at least 5 differentially expressed genes and with an adjusted p-value ≤ 0.05 are included in the table. Out of a total of 506 differentially expressed genes, 163 and 343 genes were upregulated in VD and CS with labor respectively.

*VD* vaginal delivery; *CS* Cesarean section delivery.

### CS without labor vs. vaginal delivery

Samples from CS without labor (N = 83) vs. VD (N = 181) were compared. Differential gene expression analysis resulted in 41 genes at FDR ≤ 0.05 and log_2_(fold-change) ≥ 0.5 (Fig. 1C, Supplementary Table S4). Of these, 26 (63%) were upregulated in VD and 294 (37%) were downregulated. Table 3 lists the pathways obtained with at least 5 differentially expressed genes (separated by positive or negative fold change) in a pathway. Antimicrobial peptides and neutrophil degranulation were the top pathways for genes upregulated in VD. PI3 was the top gene upregulated in VD. No significant pathways were found for genes upregulated in CS without labor. No genes were found to be differentially expressed in an independent analysis of prenatal samples from these mothers (lowest FDR > 0.99).

**Table 3:** Top pathways for differentially expressed genes in CS without labor vs. vaginal delivery.

Pathway	Adjusted p-value	Group with upregulation	Genes
Neutrophil degranulation	1.14 × 10^–14^	VD	MS4A3;DEFA4;AZU1;DEFA1;RNASE3;MPO;C3;CHIT1;SLPI;CEACAM6;TCN1;LCN2;PRTN3; CTSG;CEACAM8;ELANE;LTF
Antimicrobial peptides	3.01 × 10^–12^	VD	DEFA4;LCN2;DEFA3;PRTN3;CTSG;DEFA1;PI3;RNASE3;ELANE;LTF
Innate Immune System	1.76 × 10^–11^	VD	MS4A3;DEFA4;DEFA3;AZU1;DEFA1;RNASE3;MPO;C3;CHIT1;SLPI;CEACAM6;TCN1;LCN2; PRTN3;CTSG;PI3;CEACAM8;ELANE;LTF
Extracellular matrix organization	0.004	VD	COL17A1;CEACAM6;PRTN3;CTSG;CEACAM8; ELANE

All pathways containing at least 5 differentially expressed genes and with an adjusted p-value ≤ 0.05 are included in the table. Out of a total of 41 differentially expressed genes, 26 and 15 genes were upregulated in VD and CS without labor respectively.

*VD* vaginal delivery; *CS* Cesarean section delivery.

### CS with labor vs. without labor

Samples from CS with labor (N = 60) vs. CS without labor (N = 83) were compared. Differential gene expression analysis resulted in 263 genes at FDR ≤ 0.05 and log_2_(fold-change) ≥ 0.5 (Fig. 1D, Supplementary Table S5). Of these 263 genes, 60 (23%) were upregulated in CS without labor and 203 (77%) were upregulated in CS with labor. Table 4 lists immune pathways including interferon signaling and Cytokine signaling genes which were downregulated, and neutrophil degranulation and interleukin signaling genes, which were upregulated in CS with labor. No genes were found to be differentially expressed in the corresponding prenatal samples for the two groups (lowest FDR > 0.99).

**Table 4:** Top pathways for differentially expressed genes in CS with labor vs. CS without labor.

Pathway	Adjusted p-value	Group with upregulation	Genes
Neutrophil degranulation	4.19 × 10^–12^	CS with labor	SIGLEC9;HP;SLC2A3;GPR84;RETN;FCAR; PLAC8;TRPM2;HK3;CLEC5A;STOM; S100A12;CD59;GYG1;CD55;CD177; SERPINB1;FCER1G;CR1;ARG1;GPR97; C19orf59;ATP11B;MMP8;RNASE2; MAPK14;MMP9;CKAP4;CEACAM1; CLEC4D;TCN1;LCN2;S100P;CYSTM1; FOLR3;S100A9;CD44;S100A8
Interleukin-4 and Interleukin-13 signaling	0.004	CS with labor	SOCS3;ANXA1;HGF;LCN2;OSM;RHOU; MMP9
Innate immune system	0.006	CS with labor	SIGLEC9;HP;SLC2A3;NLRC4;GPR84;RETN;FCAR;PLAC8;TRPM2;HK3;CLEC5A;STOM; S100A12;CD59;FCGR1A;GYG1;CD55; ATP6V1C1;MAP2K6;CD177;SERPINB1; FCER1G;CR1;MYO10;ARG1;GPR97; C19orf59;ATP11B;MMP8;RNASE2; MAPK14;MMP9;CKAP4;CEACAM1; CLEC4D;TCN1;LCN2;S100P;CYSTM1; FOLR3;S100A9;CD44;S100A8
Interferon SIGNALING	7.55 × 10^–15^	CS without labor	GBP5;RSAD2;MX1;IFI6;ISG15;IFIT1; USP18;IFIT3;OASL;IFIT2;HERC5;OAS3; XAF1;GBP1
Interferon alpha/beta signaling	7.55 × 10^–15^	CS without labor	RSAD2;OAS3;MX1;IFI6;ISG15;XAF1;IFIT1;USP18;IFIT3;OASL;IFIT2
Cytokine signaling in immune system	1.10 × 10^–10^	CS without labor	GBP5;RSAD2;MX1;ALOX15;IFI6;ISG15; IFIT1;USP18;IFIT3;OASL;IFIT2;HERC5; OAS3;XAF1;GBP1
Antiviral mechanism by IFN-stimulated genes	1.39 × 10^–6^	CS without labor	HERC5;OAS3;MX1;ISG15;IFIT1;USP18; OASL

All pathways containing at least 5 differentially expressed genes and with an adjusted p-value ≤ 0.05 are included in the table. Out of a total of 263 differentially expressed genes, 203 and 60 genes were upregulated in CS with or without labor respectively.

*VD* vaginal delivery; *CS* Cesarean section delivery.

### Differentially expressed genes among all 3 groups

**Figure 2:**
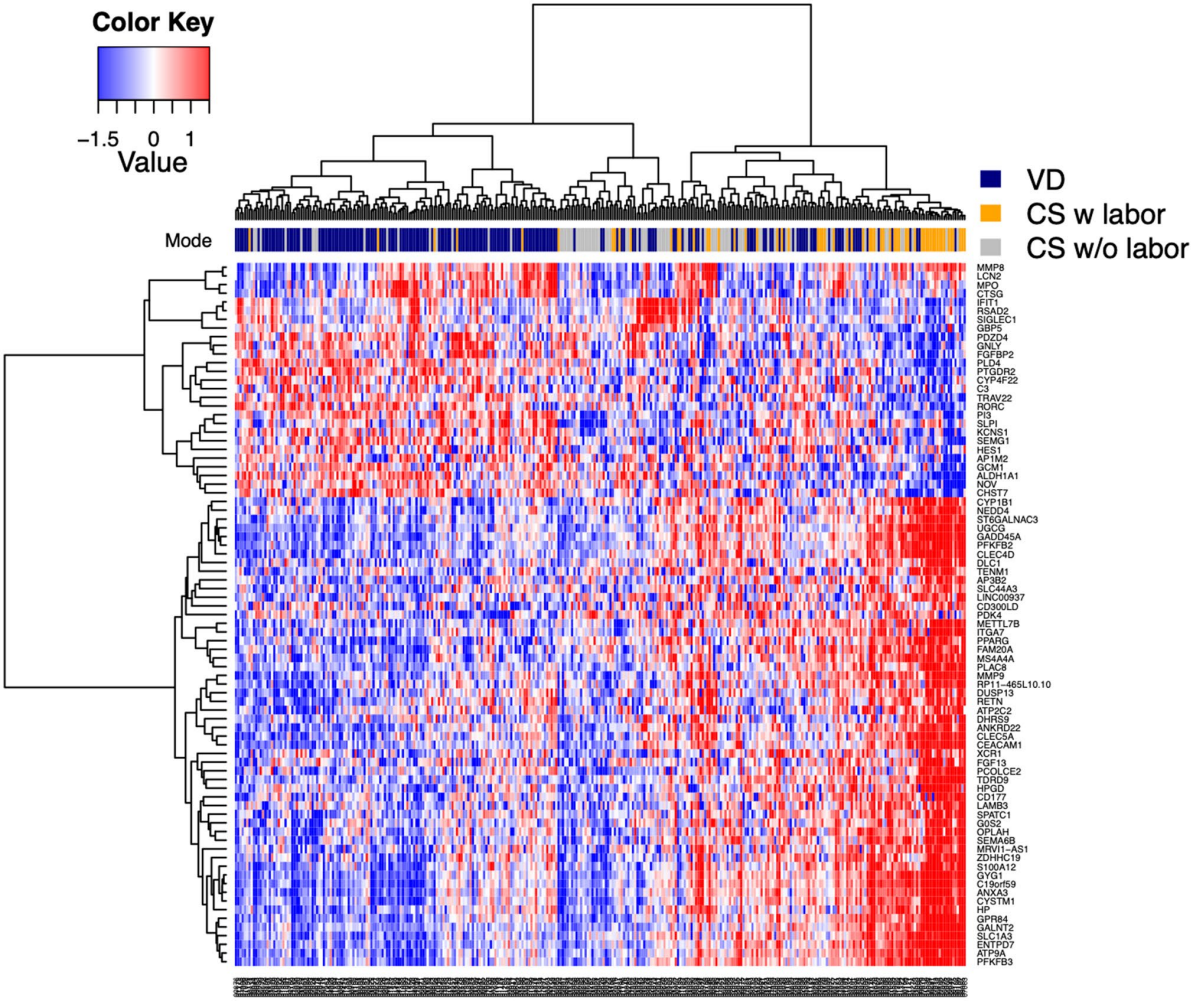
Genes differentially expressed across VD and CS with or without labor. Heatmap shows 81 significantly differentially expressed genes across all 3 delivery modes (VD, CS with labor, CS without labor). The top 50 DEGs (defined as absolute log_2_(fold-change) ≥ 0.5 and FDR ≤ 0.05) with highest fold change in either direction were selected for VD vs. CS with labor and CS with labor vs. CS without labor. All 41 DEGs were selected for VD vs. CS without labor. These 241 genes were intersected with 2,739 genes with Likelihood Ratio Test adjusted p-value < 0.001. This led to 81 genes used for clustering samples across the three delivery modes. Colors above the heatmap indicate delivery mode. Each row of the heatmap represents the z-score transformed normalized expression values of one differentially expressed gene across the subjects (blue, low expression; red, high expression). VD N = 181, CS with labor N = 60, CS without labor N = 83. *FDR* false discovery rate, *FC* fold-change, *CS* cesarean section, *VD* vaginal delivery, *DEG* differentially expressed gene.

**Figure 3:**
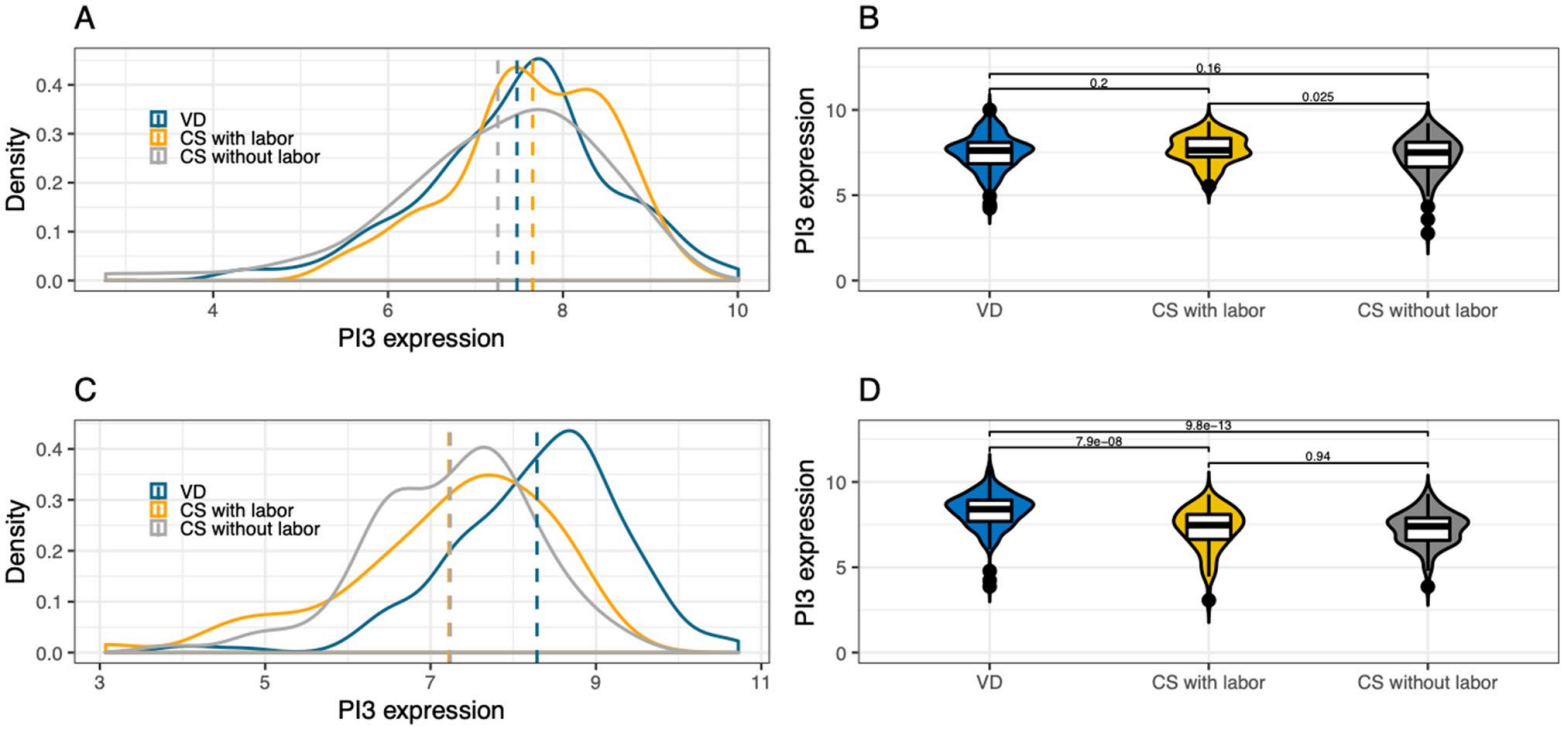
PI3 gene expression in prenatal and delivery samples across delivery modes. The plots show distribution of log of normalized counts per million (CPM). (**A**) PI3 expression density distribution across delivery modes in prenatal samples. (**B**) Violin plots with PI3 expression across delivery modes in prenatal samples. Student’s t-test p-values are included for each pairwise comparison. (**C**) PI3 expression density distribution across delivery modes in delivery samples. (**D**) Violin plots with PI3 expression across delivery modes in postpartum samples. Student’s t-test p-values are included for each pairwise comparison.

Likelihood Ratio Test (LRT) in the DESeq2^22^ package was used for identification of genes that are not constant across the three delivery modes. The top 50 genes with the highest fold-change in either direction were selected from DEGs obtained for the two comparisons (VD vs. CS with labor, CS with labor vs. CS without labor) and all 41 DEGs were selected for CS without labor vs. VD. These genes were then intersected with 2,739 genes found to show change in expression across delivery modes with LRT (adjusted p-value < 0.001). This led to 81 genes (Supplementary Table S6) that were used for clustering samples across the three delivery modes (Fig. 2). PI3 was among the genes upregulated in VD compared to CS with or without labor (Fig. 3). While an independent analysis of prenatal samples did not show elevated expression in mothers undergoing VD (Fig. 3A,B), PI3 expression was increased in VD compared to CS in post-delivery samples (Fig. 3C,D).

## Discussion

Differential gene expression was found between the delivery modes of VD, CS with labor, and CS without labor. Of particular interest, there is an enrichment of antimicrobial peptides (AMP) pathways in VD. PI3 gene, a known AMP, showed a twofold increase in VD (adjusted p-value < 10^–9^) compared to CS.

Genes in the AMP pathways are upregulated in VD samples compared to both CS with or without labor. AMPs are small molecular weight proteins that modulate host immune response against bacteria, viruses, and fungi^23,24^. Among the AMPs upregulated in VD compared to CS with or without labor (CTGS, DEFA1, DEFA1B, DEFA3, DEFA4, ELANE, GNLY, GZMH, LCN2, LTF, PI3, PRTN3, RNASE3, SEMG1), PI3 was the most significant in terms of adjusted p-value (< 10^–9^) and fold change (> twofold). The peptidase inhibitor 3 (PI3) gene encodes elafin and demonstrates antimicrobial and anti-inflammatory activities^25^. Elafin has been shown to play a protective role in inflammatory bowel disease^26^, asthma^27^, and has been shown to be downregulated in acute stages of acute respiratory distress syndrome^28^. Elafin has also been proposed to be a modulator of innate immunity in the lower genital tract^29^. Furthermore, elafin expression was found to be elevated in the cervix as a response to pathogens during preterm labor^30^. Given the apparent protective antimicrobial and anti-inflammatory role of AMPs, we postulate that lower expression of PI3 in CS deliveries compared with vaginal deliveries, even if transient, may lead to may lead to an inflammatory and immune cascade and play a role in the reported immune and inflammatory adverse outcomes associated with CS delivery for both mothers and offspring^1–3,6–16^; this warrants further exploration.

Other differentially expressed pathways between delivery modes involve the inflammatory and immune pathways (neutrophil degranulation, interferon signaling). The significance of these pathways also require further investigation given the known stress and inflammation that occur both with labor^31^ and with major surgery^17^.

Based on the known increased risk of chronic immune diseases in both mothers delivering by CS and their infants compared to VD, the finding of AMP upregulation, particularly PI3, in VD compared to CS has potential clinical implications. If further research confirms this association and a causal relationship is found, this could lead to the development of therapeutic interventions aimed to increase levels of AMPs in women undergoing CS.

Our main strength is that we included a large number of subjects in this cohort of pregnant women undergoing gene expression analyses from blood samples.

Because maternal blood sample collection was performed prenatally and postpartum, we were able to independently analyze samples from these two timepoints to conclude that changes in gene expression were influenced by the mode of delivery as no genes were differentially expressed across delivery modes in the prenatal samples.

A limitation of the study is that maternal prenatal blood was collected at various stages of the first and second trimesters between 12 and 27 weeks’ gestation (mean: 26.4 weeks, 95% confidence interval: 25.9–26.8). It is possible that confounding factors contributing to the reason a mother had a CS vs VD, including a pregnant woman’s medical and obstetric status and fetal status may lead to changes in gene expression. Although many confounding factors associated with delivery mode were adjusted for in the analysis (maternal age, maternal BMI, gestational age and computed ancestry), other factors were used as exclusion criteria for the analysis (multiple births, preterm births), and that no prenatal differences were seen in gene expression between mothers who then went on to have CS vs. VD, it is possible other confounding factors may have played a role in differences seen. It is known that following the rupture of membranes, there is an increased chance of ascending infections which may lead to increased AMP responses; in future studies, it may be valuable to account for if there was rupture of membranes, and duration between rupture of membranes and delivery. In addition, the study is limited due to the lack of long-term follow up of subjects to assess the development of possible adverse health outcomes.

In conclusion, differentially expressed genes were found in mothers according to delivery mode with enrichment of antimicrobial peptide pathways in vaginal delivery compared to both Cesarean section with or without labor. Further exploration is needed to confirm the differential expression seen, determine if similar changes are seen in gene expression, or gene products, in the offspring, and identify longer term epigenetic changes including associations with long-term clinical outcomes in both mother and baby given the risk of diseases associated with Cesarean section.

## Materials and methods

### Subject enrollment

This is a cross-sectional study carried out at Inova Fairfax Medical Center, Inova Health System, Falls Church, Virginia. The study was designed to identify genomic, clinical, and environmental risk factors that may enhance our understanding of health outcomes. Data were collected on 324 mothers who delivered from June 2013 to November 2014 and who had RNA sequencing available. Subjects were recruited during pregnancy at the time of their routine obstetric care visits. Only mothers with full term (37 weeks–0 days or more) singletons were included. Emergency and urgent CS deliveries due to maternal and fetal indications were excluded because gene expression would be expected to vary greatly across a variety of indications for time sensitive delivery. All participants provided written informed consent. The study was approved by the Western Institutional Review Board (WIRB#20120204) and the Inova Institutional Review Board (Inova IRB#15-1804). All experiments were performed in accordance with relevant guidelines and regulations.

### Data collection

Detailed demographic and clinical data were extracted from the medical record including: maternal age; pre-pregnancy body mass index (BMI); gestational age at delivery; delivery mode (VD or CS); if CS was performed with labor (regular uterine contractions and cervical dilation) or without labor; and indication for CS. Data were collected from maternal questionnaires completed prenatally including race and ethnicity.

### Sample collection and processing

Peripheral blood was obtained from enrolled mothers within 2 days of delivery (between 1 and 39 h). Prenatal blood samples (collected between 12 and 27 weeks’ gestation) were also available for these mothers. Blood was transferred into PaxGene (PreAnalytiX) tubes for total RNA isolation with the QiaSymphony (Qiagen) using Ambion MagMax RNA Isolation kit (Thermo Fisher Scientific) per manufacturer's protocol. RNA was purified and concentrated using a ZR-96 RNA Clean & Concentrator kit (Thermo Fisher Scientific) prior to quantification by NanoDrop spectrophotometry (Thermo Fisher Scientific). Total RNA was stored at – 80 °C until analysis.

### Sample sequencing

RNA samples were sequenced by the Illumina HiSeq2000 at Expression Analysis Inc. (Durham, North Carolina). For each sample, raw RNA reads in FASTQ format were aligned to hg19 using STAR 2.5.3a^32^. Standard quality control metrics were derived from the aligned reads using RNA-SeQC 1.1.9^33^. Gene-level and transcript-level expression quantifications were performed using RSEM 1.3.0^34^ against the Gencode 26 reference transcript annotation^35^. Finally, expression results in expected counts and transcripts per million (TPM) of each sample were combined into a single matrix using a customized Perl program.

### Differential gene expression and pathway analysis

EdgeR^36^ was used for differential gene expression and previously published guidelines were followed for the analysis^37^. Genes with low counts were filtered out following EdgeR recommendation where at least n samples should have > 10/L counts per million where L is the smallest library size and n is the number of samples in the smaller group. In summary, lowly expressed genes were filtered and library sizes were recalculated, followed by trimmed mean of M-values (TMM) normalization (calcNormFactors), dispersion estimation using the negative binomial (NB) model (estimateDisp), extension of the NB model with quasi-likelihood (QL) methods (glmQLFit), and finally, testing for differential gene expression (glmQLFTest). Whole genome sequencing (WGS) data were also available for these subjects; common single nucleotide variants from WGS were used to compute principal components for depicting ancestry. Age, pre-pregnancy BMI, RNA sequencing processing batch, gestational age (in weeks), duration between birth and sample collection, and the top 3 principal components derived from genomic sequencing were used as covariates in the analysis. In order to detect statistically significant changes across a range of magnitude, a cutoff of absolute log_2_(fold-change) ≥ 0.5 and false discovery rate (FDR) ≤ 0.05 were used for selecting differentially expressed genes^38^. R package ggplot2 was used to create volcano plots (Fig. 1), and density plots for PI3 expression across delivery modes (Fig. 3). Hierarchical clustering was performed and heatmap plots (Fig. 2) were generated using heatmap.3 R package. Pathway analysis was performed using Reactome^39^, a manually curated pathway database that contains 12,608 human reactions organized into 2282 pathways involving 11,053 proteins as of version 71. Genes were separated by positive and negative fold-change in a given delivery mode for each pair-wise differential expression analysis and provided as input to Reactome for pathway analysis.

### Statistical analysis

Comparisons of demographics and outcomes data were performed between women according to mode of delivery. Association between ethnicity and delivery mode was tested with Fisher’s Exact Test, as implemented in the R^40^ function fisher.test(). One-way analysis of variance (ANOVA) was performed using R function aov() to test for differences in age, BMI and gestational age across delivery modes. PI3 expression counts between delivery modes were compared using Student’s t-test, as implemented in R function t.test().

## Supplementary information

Supplementary Tables.

## References

[1] Sevelsted A, Stokholm J, Bonnelykke K, Bisgaard H (2015). Cesarean section and chronic immune disorders. Pediatrics.

[2] Cho CE, Norman M (2013). Cesarean section and development of the immune system in the offspring. Am. J. Obstet. Gynecol..

[3] Romero R, Korzeniewski SJ (2013). Are infants born by elective cesarean delivery without labor at risk for developing immune disorders later in life?. Am. J. Obstet. Gynecol..

[4] Nikischin W, Peter M, Oldigs HD (1997). The influence of mode of delivery on hematologic values in the umbilical vein. Gynecol. Obstet. Invest..

[5] Thilaganathan B, Meher-Homji N, Nicolaides KH (1994). Labor: an immunologically beneficial process for the neonate. Am. J. Obstet. Gynecol..

[6] Metsala J (2008). Perinatal factors and the risk of asthma in childhood: a population-based register study in Finland. Am. J. Epidemiol..

[7] Pistiner M, Gold DR, Abdulkerim H, Hoffman E, Celedón JC (2008). Birth by cesarean section, allergic rhinitis, and allergic sensitization among children with a parental history of atopy. J. Allergy Clin. Immunol..

[8] Roduit C (2009). Asthma at 8 years of age in children born by caesarean section. Thorax.

[9] Tollånes MC, Moster D, Daltveit AK, Irgens LM (2008). Cesarean Section and risk of severe childhood asthma: a population-based cohort study. J. Pediatr..

[10] Li H, Zhou Y, Liu J (2013). The impact of cesarean section on offspring overweight and obesity: a systematic review and meta-analysis. Int. J. Obes..

[11] Darmasseelane K, Hyde MJ, Santhakumaran S, Gale C, Modi N (2014). Mode of delivery and offspring body mass index, overweight and obesity in adult life: a systematic review and meta-analysis. PLoS ONE.

[12] Kuhle S, Tong OS, Woolcott CG (2015). Association between caesarean section and childhood obesity: a systematic review and meta-analysis. Obes. Rev..

[13] Cardwell CR (2008). Caesarean section is associated with an increased risk of childhood-onset type 1 diabetes mellitus: a meta-analysis of observational studies. Diabetologia.

[14] Cnattingius S (1991). Prenatal and neonatal risk factors for childhood myeloid leukemia. Cancer Epidemiol. Biomark. Prev..

[15] O’Donoghue K (2011). Pregnancy and the risk of autoimmune disease: an exploration. Chimerism.

[16] Khashan AS (2011). Pregnancy and the risk of autoimmune disease. PLoS ONE.

[17] Allen CJ (2017). Global gene expression change induced by major thoracoabdominal surgery. Ann. Surg..

[18] Schlinzig T, Johansson S, Gunnar A, Ekström T, Norman M (2009). Epigenetic modulation at birth: altered DNA-methylation in white blood cells after Caesarean section. Acta Paediatr..

[19] Whitney AR (2003). Individuality and variation in gene expression patterns in human blood. Proc. Natl. Acad. Sci. USA.

[20] Lee Y-S (2005). Molecular signature of clinical severity in recovering patients with severe acute respiratory syndrome coronavirus (SARS-CoV). BMC Genom..

[21] Chao A (2008). Analysis of functional groups of differentially expressed genes in the peripheral blood of patients with cervical cancer undergoing concurrent chemoradiation treatment. Radiat. Res..

[22] Love MI, Huber W, Anders S (2014). Moderated estimation of fold change and dispersion for RNA-seq data with DESeq2. Genome Biol..

[23] Zasloff M (2002). Antimicrobial peptides of multicellular organisms. Nature.

[24] Radek K, Gallo R (2007). Antimicrobial peptides: natural effectors of the innate immune system. Semin. Immunopathol..

[25] Roghanian A (2006). The antimicrobial/elastase inhibitor elafin regulates lung dendritic cells and adaptive immunity. Am. J. Respir. Cell Mol. Biol..

[26] Zhang W, Teng G, Wu T, Tian Y, Wang H (2017). Expression and clinical significance of elafin in inflammatory bowel disease. Inflamm. Bowel Dis..

[27] Tsai Y-S (2016). Protective effects of elafin against adult asthma. Allergy Asthma Proc..

[28] Wang Z, Beach D, Su L, Zhai R, Christiani DC (2008). A genome-wide expression analysis in blood identifies pre-elafin as a biomarker in ARDS. Am. J. Respir. Cell Mol. Biol..

[29] Stock SJ (2009). Elafin (SKALP/Trappin-2/proteinase inhibitor-3) is produced by the cervix in pregnancy and cervicovaginal levels are diminished in bacterial vaginosis. Reprod. Sci..

[30] Itaoka N (2015). Cervical expression of elafin and SLPI in pregnancy and their association with preterm labor. Am. J. Reprod. Immunol..

[31] Peng H-H (2011). The effects of labor on differential gene expression in parturient women, placentas, and fetuses at term pregnancy. Kaohsiung J. Med. Sci..

[32] Dobin A (2013). STAR: ultrafast universal RNA-seq aligner. Bioinformatics.

[33] DeLuca DS (2012). RNA-SeQC: RNA-seq metrics for quality control and process optimization. Bioinformatics.

[34] Li B, Dewey CN (2011). RSEM: accurate transcript quantification from RNA-Seq data with or without a reference genome. BMC Bioinform..

[35] Frankish A (2019). GENCODE reference annotation for the human and mouse genomes. Nucleic Acids Res..

[36] Robinson MD, McCarthy DJ, Smyth GK (2010). edgeR: a bioconductor package for differential expression analysis of digital gene expression data. Bioinformatics.

[37] Chen Y, Lun ATL, Smyth GK (2016). From reads to genes to pathways: differential expression analysis of RNA-Seq experiments using Rsubread and the edgeR quasi-likelihood pipeline. F1000Research.

[38] Dalman MR, Deeter A, Nimishakavi G, Duan ZH (2012). Fold change and p-value cutoffs significantly alter microarray interpretations. BMC Bioinform..

[39] Fabregat A (2018). The reactome pathway knowledgebase. Nucleic Acids Res..

[40] Team, R. C (2013). R: A Language and Environment for Statistical Computing.

